# Influence of Diosmin Treatment on the Level of Oxidative Stress Markers in Patients with Chronic Venous Insufficiency

**DOI:** 10.1155/2018/2561705

**Published:** 2018-08-28

**Authors:** Marcin Feldo, Michał Woźniak, Magdalena Wójciak-Kosior, Ireneusz Sowa, Agata Kot-Waśik, Justyna Aszyk, Jacek Bogucki, Tomasz Zubilewicz, Anna Bogucka-Kocka

**Affiliations:** ^1^Department of Vascular Surgery and Angiology, Medical University of Lublin, Staszica 11, 20-081 Lublin, Poland; ^2^Chair and Department of Biology and Genetics, Medical University of Lublin, W. Chodźki 4A, 20-093 Lublin, Poland; ^3^Department of Analytical Chemistry, Medical University of Lublin, Chodźki 4a, 20-093 Lublin, Poland; ^4^Department of Analytical Chemistry, Faculty of Chemistry, Gdańsk University of Technology, 11/12, Narutowicza, 80-233 Gdańsk, Poland; ^5^Department of Clinical Genetics, Medical University of Lublin, Radziwiłłowska 11, 20-080 Lublin, Poland

## Abstract

Oxidative stress plays an important role in the pathophysiology of many human disorders, while antioxidants prevent the development of various adverse symptoms. Diosmin is a natural flavonoid applied in vascular system disorders, especially in chronic venous insufficiency (CVI), and it plays a significant part in the alleviation of CVI symptoms. Due to antioxidant activity, it also has the ability to scavenge the oxygen free radicals and hence decreases the level of oxidative stress biomarkers, such as prostaglandins and their precursors—isoprostanes. In the study, the influence of diosmin treatment on the level of isoprostanes in plasma samples of patients suffering from CVI was examined. The qualitative analysis was performed using high-performance liquid chromatography with spectrometry detection (LC-MS). The statistically significant decrease of isoprostane content after 3 months of treatment was observed within the studied group; however, the most significant changes were observed in patients who smoke.

## 1. Introduction

Diosmin (diosmetin 7-O-rutinoside) is a natural flavone occurring in the Rutaceae family and is especially abundant in the pericarp of various citrus fruits [1]. It was first isolated from the leaves of *Scrophularia nodosa* L., (Scrophulariaceae) in 1925, and at present, it can also be obtained by dehydrogenation of another flavonoid—hesperidin. Diosmin has a wide range of biological properties confirmed by numerous *in vitro* and *in vivo* studies. It acts as an antioxidant, antihyperglycemic, anti-inflammatory, antimutagenic, and antiulcer agent [2–5].

Nowadays, due to its beneficial effects on the cardiovascular system, diosmin is one of the most desired natural compounds in the treatment of chronic venous insufficiency (CVI)—a progressive disorder affecting an increasing number of people every year. CVI is defined as morphological and functional abnormalities of the venous system manifested by varicose veins (structural changes in the vein wall), venous leg ulcers, oedema, or skin changes. The primary stage of the disease is the result of increased and sustained venous hypertension caused mostly by reflux due to incompetent venous valves. It increases hydrostatic pressure and the blood flow in capillaries. It prompts leucocyte adhesion to capillary endothelium and initialises an inflammatory reaction. Hence, capillaries become permeable to fluid which causes formation of oedema [6]. Clinical trials showed that diosmin decreases the level of pain and oedema in patients with CVI because it improves lymphatic drainage and supports microcirculation [7, 8]. It also increases capillary resistance, vascular tonus, and vein elasticity and reduces capillary filtration and capillary hyperpermeability [6]. Diosmin shows an anti-inflammatory effect by inhibiting the expression of proinflammatory cytokines through blocking the activation of NF-*κ*B pathways and reduction of T cell receptors [1]. The regression of symptoms like swelling, pain, the feeling of heaviness and tightness in legs, and an improvement in the quality of life of patients with CIV was noted [9].

The antioxidant activity of diosmin may also have a significant effect in the alleviation of CVI symptoms because oxygen-free radicals released during inflammation may cause tissue damage and a release of inflammatory mediators. It is known that oxidative stress plays an important role in the pathophysiology of many human disorders and prostaglandins and their analogues, isoprostanes, are considered as biomarkers of oxidative stress [10, 11]. For example, an increased level of both compounds has been reported in patients suffering from cardiovascular diseases [12].

Isoprostanes, especially F2-isoprostanes (F2-isoP), together with isofurans, are the major product of nonenzymatic lipid peroxidation of polyunsaturated fatty acids (PUFA) that takes place in the phospholipid domain of cell membranes [13]. Released isoprostanes circulate with the plasma and are finally excreted in the urine [12, 14]. As the mechanism of F2 isoprostane formation includes several intermediate steps, a large number of different isomers are formed. There are four regioisomeric series of F2-IsoP, named 5-, 8-, 12-, and 15-F2-IsoP or also iPF2*α*-VI, iPF2*α*-IV, iPF2*α*-V, and iPF2*α*-III, respectively [12]. In addition to the F2 isoprostanes formed from arachidonic acid, the other classes can be produced by free radical catalyzed peroxidation of fatty acids, such as eicosapentaenoic acid or docosahexanoic acid [14, 15].

Since the measurement of F2-IsoP and their metabolites may be useful to evaluate the effectiveness of clinical therapies applied to diminish oxidative stress associated with inflammation, the aim of the study was to assess the influence of diosmin treatment on the level of isoprostanes (8-iso-15(R)-PGF_2*α*_; 8-iso-PGF_2*α*_; 11*β*-PGF_2*α*_; 15(R)-PGF_2*α*_) in patients suffering from CVI.

## 2. Experimental

### 2.1. Reagents

The isoprostane standards including 8-*iso* prostaglandin F_2*α*_ (8*-iso*-PGF_2*α*_, **8** *-iso*P), 8-*iso*-15(R)-prostaglandin F_2*α*_, (8*-iso*-15(R)-PGF_2*α*_, 8,15-*iso*P), 11*β*-prostaglandin F_2*α*_ (11*β*-PGF_2*α*_, 11*-iso*P), 15(R)-prostaglandin F_2*α*_ (15(R)-PGF_2*α*_, 15*-iso*P), and the deuterated internal standard: 8-*iso* prostaglandin F_2*α*_ - d_4_ (8*-iso*-PGF_2*α* −_-d_4_, I.S.) were purchased from SPI-BIO (Montigny le Bretonneux, France). HPLC-MS grade acetonitrile was purchased from Sigma-Aldrich (Poland). Formic acid, hydrochloric acid, and ethyl acetate were bought from P.O.Ch (Gliwice, Poland). Ultrapure water was prepared with an HPLC system (Hydrolab, Poland).

### 2.2. Patient Selection

Patients were recruited from April 2014 to February 2015 at the Department of Vascular Surgery and Angiology, Medical University of Lublin. The study was approved by the Independent Ethics Committee. The inclusion criterion was the presence of CVI in the degree from 2 to 5 in the CEAP classification. None of the patients were treated with venoactive drugs for at least 3 months prior to the recruitment date. Patient characteristics are shown in Table 1.

### 2.3. Blood Collection

Blood was collected from patients before treatment and 3 months after treatment with diosmin. Patients received 2 × 600 mg/day of diosmin (Phlebodia, Laboratoires Innothera, Arcueil, France). Blood samples were collected in centrifuge tubes (Sarstedt Monovette EDTA KE, Germany), containing EDTA (1 mg/mL) and reduced glutathione (1 mg/mL), and immediately centrifuged at 1500 g for 10 min at 4°C (Eppendorf centrifuge 5702). The 890 *μ*L of plasma was transferred into 2 mL Eppendorf tubes which contained 100 *μ*L of butylated hydroxytoluene (200 *μ*L/mL) to prevent oxidation [16] and stored at −80°C for further analysis.

### 2.4. Isolation of Isoprostanes

Samples were prepared according to the literature [17]. The procedure was as follows: 400 *μ*L of the thawed plasma sample was transferred into glass tubes, 4 mL of water was acidified with hydrochloric acid (pH 2), and 4 mL of ethyl acetate was then added. Before extraction, deuterated internal standard, 8-isoP-d4, was added to achieve the final concentration of 100 pg/mL. The prepared mixture was shaken for 10 min and centrifuged for 4 min at 4400 rpm. The upper, organic layer was collected and extraction with ethyl acetate was repeated twice. Finally, 8 mL of the organic layer was collected and evaporated under a gentle stream of nitrogen. The dry residue was redissolved in 400 *μ*L of mobile phase and used for further LC-MS analysis.

### 2.5. LC/MS Analysis

**Figure 1:**
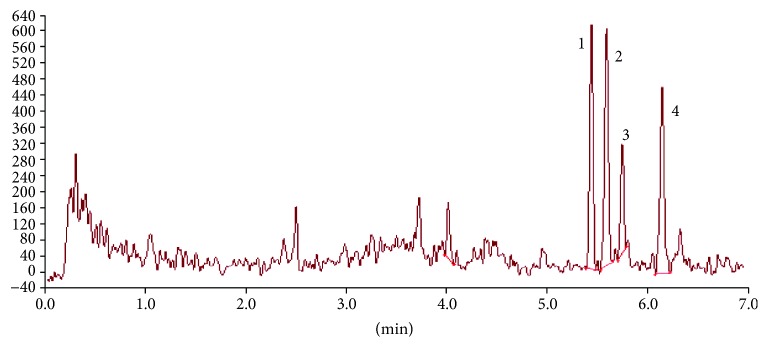
The exemplary chromatogram of plasma sample from patients with CVI. (1) 8-iso-15(R)-prostaglandin F2*α*; (2) 8-iso-prostaglandin F2*α*; (3) 11*β*-prostaglandin F2*α*; (4) 15(R)-prostaglandin F2*α*.

LC-MS 8050 Shimadzu system composed of a binary pump (NEXERA X2 LC-30 AC), a thermostat (CTO-20AC PROMINENCE COLUMN OVEN), an autosampler (NEXERA X2 SIL-30AC), and a triple quadrupole mass spectrometer (Shimadzu, Japan), equipped with an ESI source operated in the negative ion mode, was used in the study. For all the analyses, the C18 column Kinetex, Phenomenex (150 × 2.1 mm, 2.6 *μ*m) maintained at 50°C was used. 0.015% formic acid in water (A) and 0.01% formic acid in acetonitrile (B) were used as mobile phases in the following gradient: 15% of B kept for 1 min, then linear gradient from 15% to 100% B in 7 min. The flow was 0.7 mL/min, and the injection volume was 50 *μ*L. The acquisition mode used was multiple reaction monitoring (MRM). The exemplary chromatogram obtained in the above condition is presented in Figure 1. Quantification was performed using multiple reaction monitoring (MRM) transitions of m/z 353.10→193.10 for all isoprostanes, 357.20→197.10 for 8-*iso* prostaglandin F_2*α*_-d_4_ (IS). LabSolutions 5.60 SP1 software was used in data acquisition and analysis. Detailed methodology and validation were previously described in more detail [17].

### 2.6. Statistical Analysis

Statistical analysis of the results (Statistica 10 software, StatSoft Polska Sp. z o.o.) was carried out using descriptive statistics and the following parameters: group size (*N*), arithmetic mean, median, minimum and maximum value of variables, standard deviation (SD), and mean standard error. The Mann-Whitney *U* test was conducted to evaluate the significance of differences between smokers and nonsmokers.

## 3. Results and Discussion

The measurement of the F2-isoprostane level allows an assessment of the oxidative stress status *in vivo*, and it provides information on the role of the oxidative stress in the pathogenesis of various human diseases. There are some literature data on the relationship between isoprostanes in urine or blood and cardiovascular disease (CVD) [18]. The increased content of the compounds when compared to the control group were observed in patients, e.g., with hypertrophic cardiomyopathy (35.4 vs. 29.9 pg/mL, respectively) [19] and atherosclerotic lesions (75.9 and 11.7 pg/mg, respectively) [20].

Diosmin, like most flavonoids, has the ability to scavenge oxygen-free radicals [21], and hence, it should also decrease oxidative stress markers such as isoprostanes. The aim of this study was to assess the influence of 3 months of treatment with diosmin (2 × 600 mg) on the isoprostane level in patients suffering from CVI. Four compounds were monitored: 8-iso-15(R)-PGF2*α*, 8-iso-PGF2*α*, 11*β*-PGF2*α*, and 15(R)-PGF2*α*.

**Figure 2:**
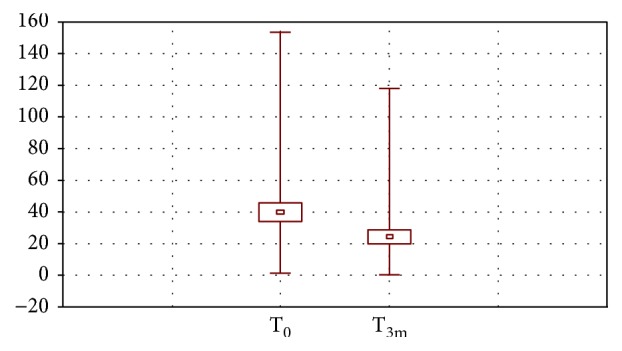
Mean (±SEM), minimal and maximal content of isoprostanes in plasma before (T_0_) and after 3 months (T_3m_) of treatment with diosmin.

Generally, a high variation of isoprostane content was observed in the studied group both before (T_0_) and after (T_3m_) diosmin treatment (Figure 2). The values were in the range 0.1–153.8 pg/mL (median 24.7 pg/mL) and 0.1–118.1 pg/mL (median 12.7 pg/mL), respectively. The mean T_0_ value was 39.65 ± 42.1, and after 3 months, it decreased to 23.97 ± 31.3 pg/mL (the difference was statistically significant). The most significant changes of isoprostane content were noted for 11*β*-PGF2*α* and 15(R)-PGF2*α* (Table 2). The other physiological parameters increasing the risk of CVI, such as total cholesterol, HDL, LDL, triglyceride fraction, and the body mass index (BMI), were also monitored in patients (Table 3); however, no effect after diosmin treatment was observed. Analysing the data from individual subjects (Figure S1), a statistically significant lowering of isoprostane content was noted in 25 patients, and the changes were statistically insignificant or an increase up to 35 pg/mL was observed in 12 and 7 patients, respectively. In three patients, an abnormal level of isoprostanes was noted (above 93 pg/ml); however, it was probably the result of the additional occurrence of another disease related to oxidative stress during treatment with diosmin.

**Figure 3:**
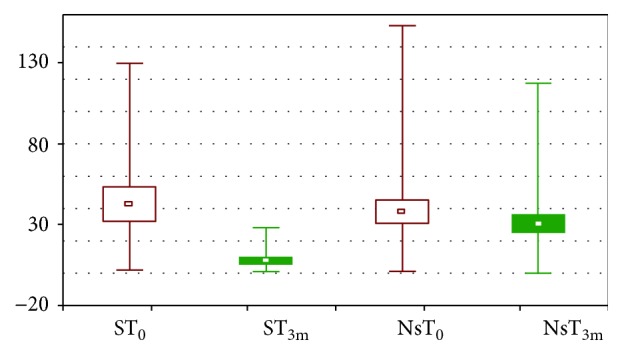
Mean (±SEM), minimal and maximal content of isoprostanes in plasma before (T_0_) and after 3 months (T_3m_) of treatment with diosmin. S: smokers; Ns: nonsmokers.

Within the investigated group of patients, 14 patients were noted to be smokers and it was expected that in this group, the initial level of isoprostanes would be higher than in nonsmokers. It was proved that smoking increases oxidative stress because of oxidants occurring in cigarette smoke or reactive oxygen species generated by phagocytic cell activation [22]. Moreover, nicotine suppresses the activity of antioxidant enzymes, such as glutathione peroxidases (GPx), superoxide dismutase (SOD), and glutathione reductase (GR). Morrow et al. [23] observed that the level of free and esterified F2-isoprostanes in plasma were ca. twofold higher in smokers when compared to nonsmokers, and two weeks of abstinence allowed a decrease in the content of isoprostanes. Surprisingly, in our study, the difference in both investigated groups before diosmin treatment was not statistically significant; the mean content of isoprostanes was 42.96 ± 40.3 and 38.25 ± 43.3 pg/mL, respectively. There were also no statistically significant differences in physiological parameters such as cholesterol level and BMI between smoking and nonsmoking group of patients. However, the percentage of patients with extremely high level of isoprostanes (above 50 pg/mL) was higher in smokers than in nonsmokers (35.7 and 24.2%, respectively). After 3 months of diosmin treatment, the level of isoprostanes in smokers decreased significantly (mean value was 7.74 ± 9.9 pg/mL); in turn, only a slight (statistically nonsignificant) decrease was observed in nonsmokers. The comparison of results obtained in both groups before and after 3 months of diosmin treatment is shown in Figure 3. The ability to reduce the nicotine oxidative stress was observed also in animal models for the other flavonoids like silymarin and naringenin [24], as well as for antioxidants, such as vitamin E.

### 3.1. Study limitation

The study has several limitations, with the most important being the relatively small number of patients and the fact that the CVI study group was heterogeneous (comorbidity such as diabetes, coronary disease, and hypertension). Thus, the influence of patient comorbidities on the final study results was difficult to estimate. However, there was no statistically significant differences in plasma level of isoprostanes between group with and without comorbidities. The fact that all included patients were treated according to protocol and all data were studied statistically are strengths of the investigations.

## 4. Conclusions

In our study, the change in the isoprostane level before and after 3 months of treatment with diosmin in patients with chronic venous insufficiency was investigated. A high variation of isoprostane content was observed within the studied group. The mean T_0_ value was 39.65 ± 42.1, and after 3 months, it decreased to 23.97 ± 31.3 pg/mL (the difference was statistically significant). The most significant changes were observed in patients who smoke, where the level of isoprostanes decreased from 42.96 to 7.74 pg/mL. The obtained results confirmed the ability of diosmin to alleviate oxidative stress.

## Figures and Tables

**(a):** 

Age (years) Mean (±SD)	61 (±10.4)
Sex (m/f) [%]	57.4/42.6
Smokers [%]	29.8

**(b):** 

CEAP class [%]			
2	3	4	5
48.9	36.2	10.6	4.3

Venous insufficiency operation [%]			
Hypertension	Coronary heart disease	Diabetes	Heart attack
44.7	6.4	8.5	4.3

**(c):** 

Cardiological treatment [%]		
*β*-blockers	ASA	Statins
10.6	31.9	10.6

**(d):** 

Antidiabetes treatment [%]	8.5

**Table 2:** The level of isoprostanes (pg/ml) in patients before (T_0_) and after 3 months of treatment with diosmin (T_3m_).

	8-iso-15(R)-PGF2*α*	8-iso-PGF2*α*	11*β*-PGF2*α*	15(R)-PGF2*α*	Total
T_0_	3.09	6.61	10.54	19.41	39.65
T_3m_	4.28	5.91	1.29	12.50	23.97

**Table 3:** The selected physiological parameters in patients before (T_0_) and after 3 months of treatment with diosmin (T_3m_) (±SD).

Parameter	T_0_	T_3m_
Total cholesterol [mg/dl]	205.4 (±29.3)	202.4 (1 ± 6.5)
HDL [mg/dl]	41.9 (±8.1)	42.6 (±10.7)
LDL [mg/dl]	102.8 (±23.5)	103.1 (±19.1)
Triglycerides [mg/dl]	118.85 (±23.4)	110.4 (±32.6)
BMI	25.83 (±2.1)	25.8 (±2.3)

## Data Availability

The detailed patients data used to support the findings of this study have been deposited in the Department of Vascular Surgery and Angiology, Medical University of Lublin, Staszica 11, 20-081 Lublin, Poland. The data used to support the findings of this study are available from the corresponding author upon request.

## Supplementary Materials

Supplementary Materials Figure S1: the content of total isoprostane level in individual patient within the investigated group. (1–14: smokers; 15–48: nonsmokers; blue colour: before treatment with diosmin; red colour: after 3 months of treatment with diosmin).
