# Supplementary material for: Influence of Diosmin Treatment on the Level of Oxidative Stress Markers in Patients with Chronic Venous Insufficiency
Source: Oxid Med Cell Longev. 2018 Aug 28;2018:2561705. doi: 10.1155/2018/2561705 (PMC6136498; doi:10.1155/2018/2561705)
Supplement: Supplementary Materials — Figure S1: the content of total isoprostane level in individual patient within the investigated group. (1–14: smokers; 15–48: nonsmokers; blue colour: before treatment with diosmin; red colour: after 3 months of treatment with diosmin). [file 2561705.f1.docx]

**Influence of diosmin treatment on the level of oxidative stress markers in patients with chronic venous insufficiency**

Marcin Feldo^1*^, Michał Woźniak^2^, Magdalena Wójciak-Kosior^3^, Ireneusz Sowa^3^, Agata Kot-Waśnik^4^, Jusyna Aszyk^4^, Jacek Bogucki^5^, Tomasz Zubilewicz^1^, Anna Bogucka-Kocka^2^

*^1^ Department of Vascular Surgery, Medical University of Lublin, Staszica 11, 20-081 Lublin, Poland*

*^2^Chair and Department of Biology and Genetic Medical University of Lublin, W. Chodźki 4A, 20-093 Lublin, Polanda*

*^3^ Department of Analytical Chemistry, Medical University of Lublin, Chodźki 4a, 20-093 Lublin, Poland*

*^4^ Department of Analytical Chemistry, Faculty of Chemistry, Gdansk University of Technology, 11/12, Narutowicza Street, 80-233 Gdańsk, Poland*

*^5^ Department of Clinical Genetics, Medical University of Lublin, Lublin, Poland, Radziwiłłowska 11, 20-080 Lublin*

^*^ corresponding author: [martinf@interia.pl](mailto:martinf@interia.pl)

Fig. S1. The content of total isoprostanes level in individual patient within the investigated group. (no 1-14 smokers, 15-48 non-smokers, blue colour - before treatment with diosmin, red colour - after 3 months of treatment with diosmin).
